# Microvascular response to cold stress in healthy humans

**DOI:** 10.1186/cc12151

**Published:** 2013-03-19

**Authors:** A Pranskunas, R Rasimaviciute, J Paltanaviciute, L Daniuseviciute, V Pilvinis, M Brazaitis

**Affiliations:** 1 Lithuanian University of Health Sciences, Kaunas, Lithuania; 2 Lithuanian Sports University, Kaunas, Lithuania

## Introduction

Cold exposure can be adapted for exercise or therapeutic purposes, but its impact on microcirculation in healthy humans has not been well defined. We hypothesize that whole body cold stress may impair microcirculation.

## Methods

Seven volunteers were recruited for the water immersion procedure. During the cooling protocol the volunteers every 20 minutes of immersion were asked to step out from the bath and rest for 10 minutes in a room environment and then return to the water bath for the next 20 minutes of immersion. This head-out immersion procedure in bath water at 14°C continued until the rectal temperature was dropped to 35.5°C or the time of 180 minutes was terminated. Maximum cold water immersion time was 120 minutes. Before, at the end of whole body cooling and 1 hour after cooling was ended, systemic hemodynamics and direct *in vivo *observation of the sublingual microcirculation were obtained with sidestream dark-field imaging. Assessment of microcirculatory parameters of convective oxygen transport (microvascular flow index (MFI), proportion of perfused vessels (PPV)), and diffusion distance (perfused vessel density (PVD) and total vessel density (TVD)) was done using a semiquantitative method.

## Results

During cooling and 1 hour after cooling was ended, a significant increase in cardiac output (*P *= 0.028 and *P *= 0.043) was observed, but there were no changes in heart rate or mean arterial pressure in comparison with baseline variables. There were no significant changes in PPV, MFI, PVD and TVD of small vessels in comparison with baseline variables during all observational time.

## Conclusion

Defined cold exposure had no effect on the micro-circulation.

